# The development and application of chimeric antigen receptor natural killer (CAR-NK) cells for cancer therapy: current state, challenges and emerging therapeutic advances

**DOI:** 10.1186/s40164-024-00583-7

**Published:** 2024-12-04

**Authors:** Pin Yao, Ya-Guang Liu, Gang Huang, Liangchun Hao, Runan Wang

**Affiliations:** 1 Department of Health Management, Shengjing Hospital of China Medical University, Shenyang, 110004 Liaoning China; 2 Department of Ultrasound, Shengjing Hospital of China Medical University, Shenyang, 110004 Liaoning China; 3 Department of Pathology and Laboratory Medicine, University of Texas Health Science Center at San Antonio, San Antonio, TX 78229 USA; 4 Department of Cell Systems and Anatomy, University of Texas Health Science Center at San Antonio, San Antonio, TX 78229 USA; 5 Department of Pediatrics, Shengjing Hospital of China Medical University, No.36, Sanhao Street, Shenyang, 110004 Liaoning China

**Keywords:** Chimeric antigen receptor (CAR), Single-chain variable fragment (scFv), Natural killer cells, Major histocompatibility complex (MHC), Tumor microenvironment (TME), Induced pluripotent stem cell (iPSC)

## Abstract

Immunotherapy has transformed the landscape of cancer treatment, with chimeric antigen receptor (CAR)-engineered T (CAR-T) cell therapy emerging as a front runner in addressing some hematological malignancies. Despite its considerable efficacy, the occurrence of severe adverse effects associated with CAR-T cell therapy has limited their scope and prompted the exploration of alternative therapeutic strategies. Natural killer (NK) cells, characterized by both their innate cytotoxicity and ability to lyse target cells without the constraint of peptide specificity conferred by a major histocompatibility complex (MHC), have similarly garnered attention as a viable immunotherapy. As such, another therapeutic approach has recently emerged that seeks to combine the continued success of CAR-T cell therapy with the flexibility of NK cells. Clinical trials involving CAR-engineered NK (CAR-NK) cell therapy have exhibited promising efficacy with fewer deleterious side effects. This review aims to provide a concise overview of the cellular and molecular basis of NK cell biology, facilitating a better understanding of advancements in CAR design and manufacturing. The focus is on current approaches and strategies employed in CAR-NK cell development, exploring at both preclinical and clinical settings. We will reflect upon the achievements, advantages, and challenges intrinsic to CAR-NK cell therapy. Anticipating the maturation of CAR-NK cell therapy technology, we foresee its encouraging prospects for a broader range of cancer patients and other conditions. It is our belief that this CAR-NK progress will bring us closer to making significant strides in the treatment of refractory and recurrent cancers, as well as other immune-mediated disorders.

## Introduction

Immunotherapy has revolutionized cancer treatment. Immune checkpoint inhibitors (ICIs) and adoptive cell transfer (ACT) represent key forms of immunotherapy that have yielded durable clinical responses. While ICIs have delivered impressive results, their use does not represent a universal victory for all cancer patients. In cases of metastatic disease, most responses are not complete. Additionally, the impact of immune checkpoint inhibitors in the treatment of solid tumors has shown limitations [1]. Adoptive cell transfer (ACT) involves the use of either natural host cells displaying inherent antitumor reactivity or host cells that have been genetically engineered with antitumor T cell receptors (TCRs) or chimeric antigen receptors (CARs) [2]. CARs were created by fusing antibody V domains with T-cell receptor domains, providing T cells with customizable and MHC-independent specificity [3–5]. This versatile CAR platform has been vastly improved since its initial fruition, demonstrating promising clinical effects on hematological malignancies previously believed to be untreatable [6–8]. It is sufficient to say that this type of therapeutic is driving rapid technological advancements, clinical applications, and human clinical trials [6–8]. Four anti-CD19 CAR-T cell products and two anti-BCMA CAR-T cell products have been approved by the US Food and Drug Administration (FDA) for clinical applications [6, 7]. In the CD19 CAR-T cell multicenter trials, complete response (CR) rates of 40–74% were observed in patients with R/R B-cell malignancies. Complete remission of various B-cell malignancies lasting ≥ 3 years was observed in 51% of evaluated cases following anti-CD19 CAR-T cell treatments with some remissions having persisted for up to 9 years and counting [7, 8]. An anti-BCMA CAR-T treatment meta-analysis of 21 relevant trials with 761 relapsed/refractory multiple myeloma (RRMM) patients reported the CR rate of 34–54%, median progression free survival (PFS) of 8.77 months, and the median duration of response (DOR) as 10.32 months [9]. CAR-T cell resistance has persisted in majority of patients with hematological malignancies and solid tumors [6]. Current treatment with CAR-T cells has been linked to significant toxic effects, notably cytokine release syndrome, neurotoxicity and hemophagocytic lymphohistiocytosis or macrophage activation syndrome (HLH/MAS) [10–13]. In order to solve current challenges and limitations, novel CAR-T cell platforms and other CAR-based cells therapies have been introduced as alternative options or complementary to CAR-T cell therapy in cancer and non-malignant devastating diseases [14–20].

Natural Killer (NK) cells are specialized immune effector cells that play a crucial role in the immune response against abnormal cells. Notably, NK cells have demonstrated positive effects and safety in most clinical trials conducted thus far. Immunotherapies centered around NK cells stand out as among the most promising treatments currently in development for addressing previously incurable forms of leukemia and various other types of cancer [21]. The therapeutic efficiency of non-engineered NK cells is often suboptimal, particularly in the context of solid tumors. Significant efforts are underway for the engineering and innovation of NK cell-based immunotherapy [22]. CAR-engineered NK cells, incorporating innovative strategies, have shown promising results in both preclinical and clinical settings, demonstrating efficacy and reducing deleterious effects [23, 24]. The studies in the CAR-NK cell field represent the forefront of cancer immunotherapy, showcasing advances in new technology and knowledge within the realm of biomedicine. Many informative reviews have outlined the foundational and clinical advancements in CAR-NK cell development [15, 25–31]. Our review aims to provide a detailed examination of the design principles and concrete rationales underpinning CAR-NK cell immunotherapy. Our focus is directed toward the latest approaches and strategies in CAR-NK cell engineering, along with significant advancements achieved in preclinical and clinical research. By providing these insights, we hope to provide researchers and clinicians with practical knowledge of the field and harness the therapeutic potential of CAR-NK immunotherapy.

## NK cell biology

### Characterization and heterogeneity of NK cells

**Fig. 1:**
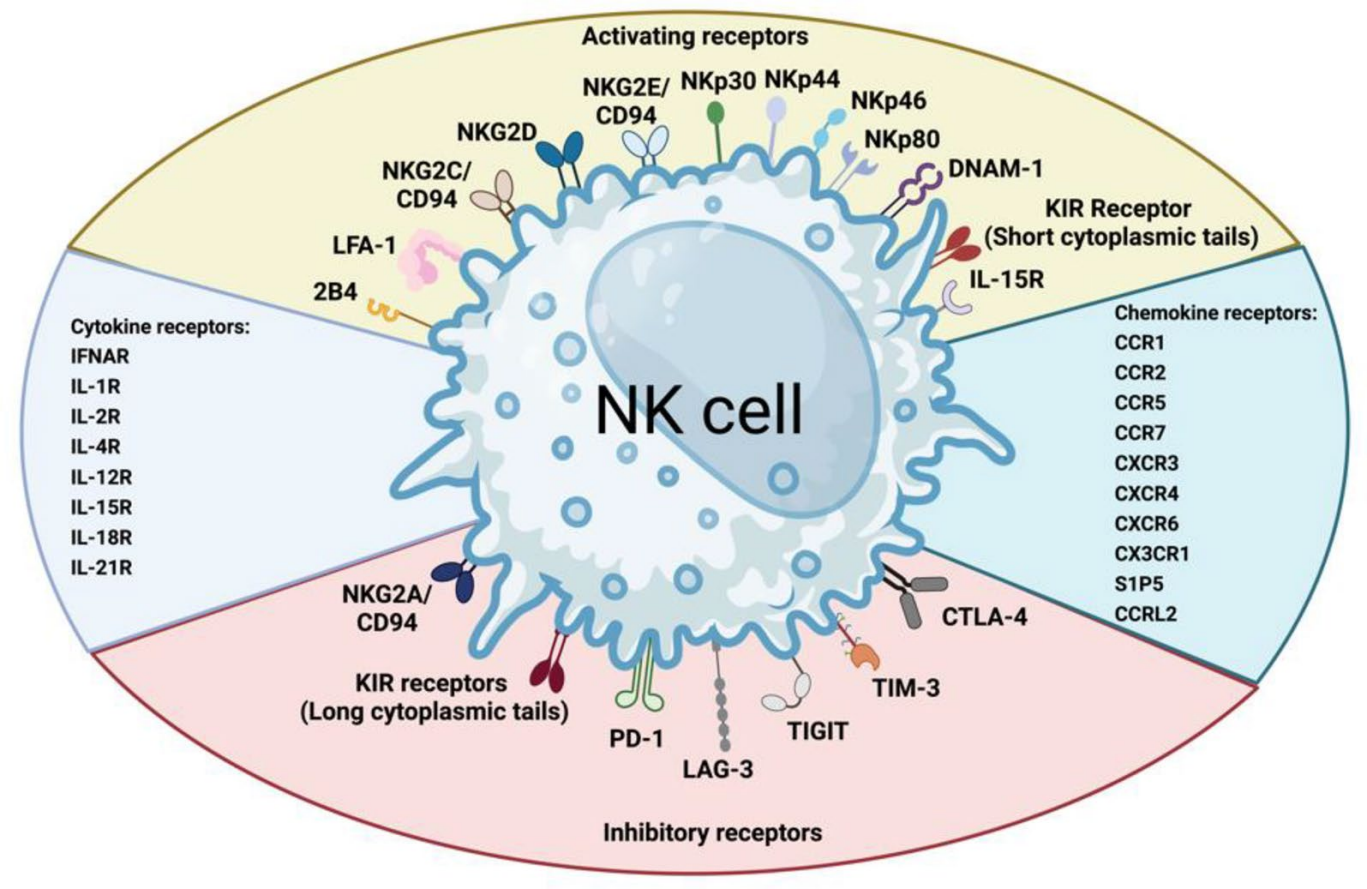
Major characteristic receptors on human NK cell. Created with BioRender.com.

NK cells are innate immune effector cells, NK cells develop from CD34+ progenitor cells in the bone marrow. However, it remains unclear whether they originate from a distinct set of precursor cells or from multipotent progenitors that also give rise to T lymphocytes, B lymphocytes, and myeloid cells [32]. In contrast to T cells and NKT cells, NK cells do not express the clonotypic TCR and the associated CD3 complex responsible for antigen recognition and signal transduction [29]. NK cells are characterized by relative expression of surface markers CD56 and CD16. NK cells display substantial phenotypic heterogeneity, as revealed by high-parameter cytometry and single-cell proteo-genomics technologies. This diversity in phenotype corresponds to varying functional properties among NK cells [33]. Human NK cell development is often depicted as a linear model, commencing with the earliest precursor (stage 1) and progressing towards terminally mature CD56^dim^ CD16+ (stage 5) or CD56^dim^ CD16+CD57+ (stage 6) cells. This sequential model outlines the developmental stages through which NK cells undergo maturation, acquiring specific phenotypic markers along the way [34]. The successive stages in natural killer cell maturation are defined by escalating lineage restriction, accompanied by alterations in phenotype and function [34]. Human NK cells express major characteristic receptors (Fig. 1) [35]. The mechanisms underlying human natural killer cell development have not been fully elucidated, particularly regarding the signals responsible for driving the spatial localization and maturation of natural killer cells [34]. IL-15 plays a pivotal role in mediating essential signals for homing, function, maturation, proliferation, and survival throughout the lifespan of NK cells [36]. IL-7 serves as a regulator of immature NK cell survival and homeostasis. However, it is not independently sufficient or required for terminal NK cell maturation in the absence of other cytokines, such as IL-15 or IL-2 [34]. IL-12 serves as a stimulator that promotes the production of IFN-γ, collaborating with IL-18 to augment the cytotoxicity of NK cells [37]. CD56^dim^ NK cells acquiring CD57 form a terminally mature subset with a greater killing capacity, the CD57^+^ NK cells constitute a heterogeneous population with variable cell surface receptors and transcriptional divergence [38, 39]. Of clinical importance, uneducated human NK cells can be activated with IL-12, IL-15, and IL-18, overcoming their baseline hyporesponsiveness. This activation boosts functionality, particularly in response to CD16 triggering and AML target cells [40]. Mechanically, Eomesodermin (EOMES) and T-box transcription factor 21 (T-BET) are required for sustaining mature NK cell identity and functional activity, ETS proto-oncogene 1 transcription factor (ETS1) plays an important regulator of human NK cell terminal differentiation [41–43]. NK cell terminal differentiation is a continuous process that can adapt in response to stresses in niche from a broad range of virally infected, stressed and transformed cells, characterizing with progressive phenotypic changes and defined effector signatures [44]. These terminally differentiated subsets mediate immunosurveillance through diverse peripheral tissue sites [45].

### Homing and trafficking of NK cells in the tumor microenvironment (TME)

**Fig. 2:**
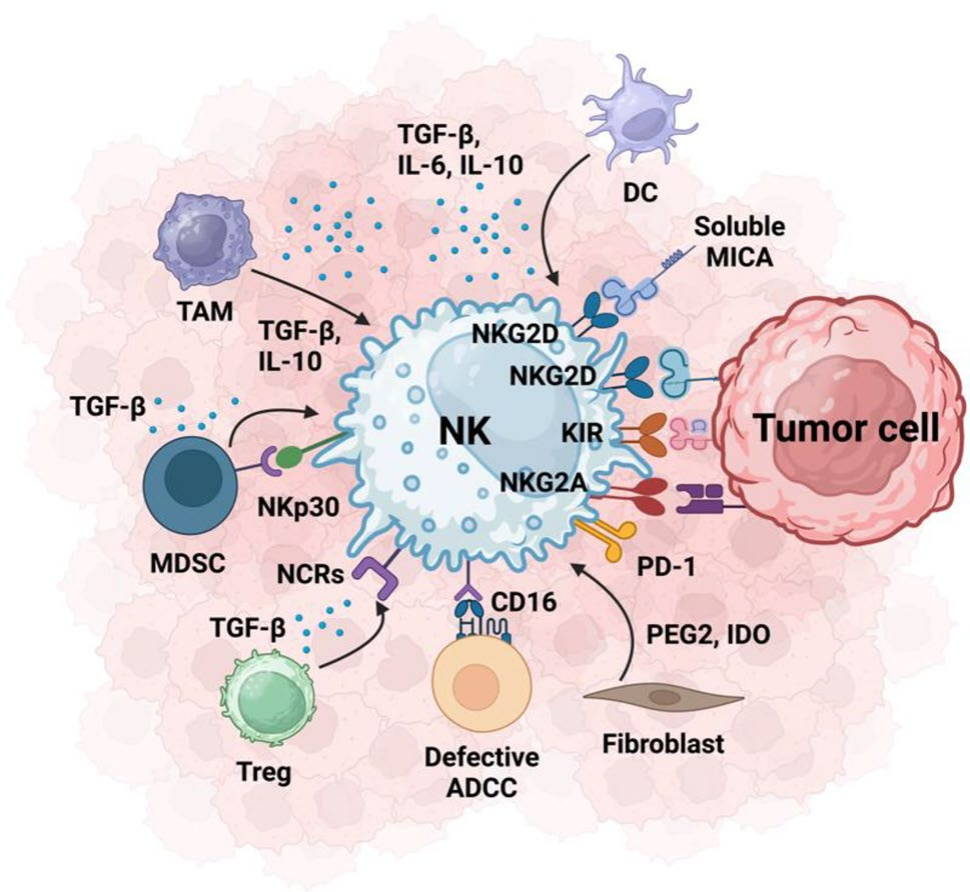
NK cells interact with other cells in TME. Created with BioRender.com

NK cells are frequently present in TME of human tumors, encompassing primary tumors, metastases, and tumor-infiltrated lymph nodes. NK cells in TME commonly experience exhaustion and metabolic impairment [46]. Intratumoral NK cells in most solid tumors are often dysfunctional and even promote tumor growth under certain states [47]. The presence of CD56^dim^ NK cells in TME has been correlated with superior survival in head and neck squamous cell carcinoma (HNSCC) [48] In the TME, peripheral NK cells undergo differentiation into two distinct subsets: hyporesponsive NK cells and highly active NK cells. The highly active NK cell phenotype exhibits potent antitumor properties [49]. NK cell trafficking and homing are intricately regulated by a combination of cell-intrinsic factors, such as transcriptional factors, cell-extrinsic factors (including integrins, selectins, chemokines and their receptors, signals induced by cytokines, sphingosine-1-phosphate (S1P), etc.), and the surrounding cellular microenvironment [50]. Studies indicate that NK cells possess the capability to efficiently migrate to tumor sites through chemokine signaling, with C-X-C Motif Chemokine Receptor 3 (CXCR3) playing a particularly crucial role in the localization of NK cells into tumors [51]. Activated NK cells in the TME not only have the capacity to directly lyse tumor cells, generating additional tumor antigens, but also function as a specialized source of chemokines and cytokines to recruit other immune cells (Fig. 2). This dual role contributes to the immune inflammatory response against tumors [52]. Additionally, circulating NK cells have demonstrated the ability to impede disease progression by inhibiting tumor metastasis [52].

### Molecular basis of NK cell functionality

Despite the absence of TCR, NK cells maintain the expression of the ζ chain derived from the CD3 signaling complex. Without prior antigen exposure, NK cells employ four distinct mechanisms to recognize target cells: (1) natural cytotoxicity; (2) antibody-dependent cell cytotoxicity (ADCC) mediated by CD16 (a low-affinity Fc receptor for IgG); (3) engagement of tumor necrosis factor (TNF)-related apoptosis-inducing ligand; and (4) activation through Fas ligand [53, 54]. While CD16-mediated ADCC has been well characterized, our current understanding of other NK cytotoxicity mechanisms, particularly those involving activating receptors and their corresponding ligands for targeting NK-susceptible cells, remains largely elusive [55]. Natural cytotoxicity is dynamically controlled by a precise balance between activating and inhibitory signaling pathways. NK cells have the ability to detect transformed tumor cells based on the expression or absence of ligands associated with cancer [56]. The efficacy of NK cells in targeting and eliminating cells is contingent upon the intricate interplay of Human Leukocyte Antigen (HLA) class I activating and inhibitory receptors expressed on their cell surface [55]. The major human activating NK cell receptors include the nature cytotoxicity receptors (NKp46, NKp44 and NKp30) that are important for NK cell natural cytotoxicity against tumors and infected cells. NKp46 and NKp30 are expressed on all human NK cells, while NKp44 expression must be induced on activated NK cells by cytokines. NKG2D and DNAX accessory molecule-1 (DNAMI-1) are activating receptors expressed by NK cells and inducing expression of cognate ligands on tumor cell surface during oncogenic insults renders target cells susceptible to immune destruction [57, 58]. DNAM-1 is a critical regulator involving NK cell education and differentiation [58].

NK cell inhibitory receptors such as Killer Ig-Like Receptor (KIR/CD158) family and the CD94/NKG2A (CD94/CD159a) heterodimer collectively orchestrate the fail-safe mechanism against NK-mediated damage to healthy cells [59]. Inhibitory KIRs are type I transmembrane receptors that are specific for polymorphic HLA-A, B and C molecules, whereas NKG2A is a type II transmembrane receptor that recognizes HLA-E. These inhibitory receptors contain ITIM motifs in their cytoplasmic tail [60]. Inhibitory checkpoints, namely PD-1, T cell immunoreceptor with Ig and ITIM domains (TIGIT), CD96, and T-cell immunoglobulin mucin family member 3 (TIM-3), play a pivotal role in preserving immune cell homeostasis. When binding to its ligands (PD-L1 and PD-L2), PD-1 may maintain peripheral tolerance but also compromises anti-tumor immunity [61, 62]. The co-inhibitory receptor TIGIT decreases NK cell cytotoxicity and is involved in NK cell exhaustion [63]. Upon engaging with CD155 expressed on target cells, CD96 has been identified as an inhibitor of mouse NK cells. Notably, interventions such as antibody blocking or the genetic knockout of CD96 have demonstrated significant therapeutic benefits, effectively restraining tumor growth and mitigating metastatic dissemination in murine model systems [64]. Blockade of TIM-3 (known as HAVCR2, Hepatitis A virus cellular receptor) results in increased NK cytotoxicity [65]. Homing receptors or molecules play a pivotal role for regulating NK cell homing and trafficking in tissue and tumors [60, 66]. The immunological synapse between NK cell and tumor cell is specialized to facilitate cytotoxic activity against tumor cell. The synapse formation may be closely related to some adhesion molecules, CD2 rapidly accumulates on the NK cell surface on initial adhesion to tumor cell during the early interactions [67], which further facilitating the formation of the firm adhesion-the lytic synapse through the involvement of key integrins such as lymphocyte function-associated antigen 1 (LFA1). Engagement of both LFA-1 and CD16 leads to polarization of the lytic machinery and degranulation in the direction of the target tumor cell [68]. Homing receptors such as CXCR3, CXC6, CCR2 and CCR5 are associated with lung-homing [69]. NK cells can enter the central nervous system (CNS) by crossing the blood–brain barrier and the choroid plexus through some chemokines (CXCL1, CCL2 and CXCL10)-associated recruitment [70]. NK cells enhance anti-tumor response by recruiting additional immune cells into the TME via the secretion of cytokines or chemokines [50]. NK cells can secrete various cytokines and chemokines to not only enhance CD8+ T cell cytotoxic response and the antigen-presenting ability of macrophages but also recruit dendritic cells into the TME [71]. High expectations for the treatment of both hematologic malignancies and solid tumors are grounded in the utilization of NK cells expressing chimeric antigen receptors (CARs) specific to tumor antigens along with NK cell characterizations in potent natural tumor cytotoxicity and distinctive homing capacity.

## CAR basic structures and their modifications for engineering CAR-NK cells

**Fig. 3:**
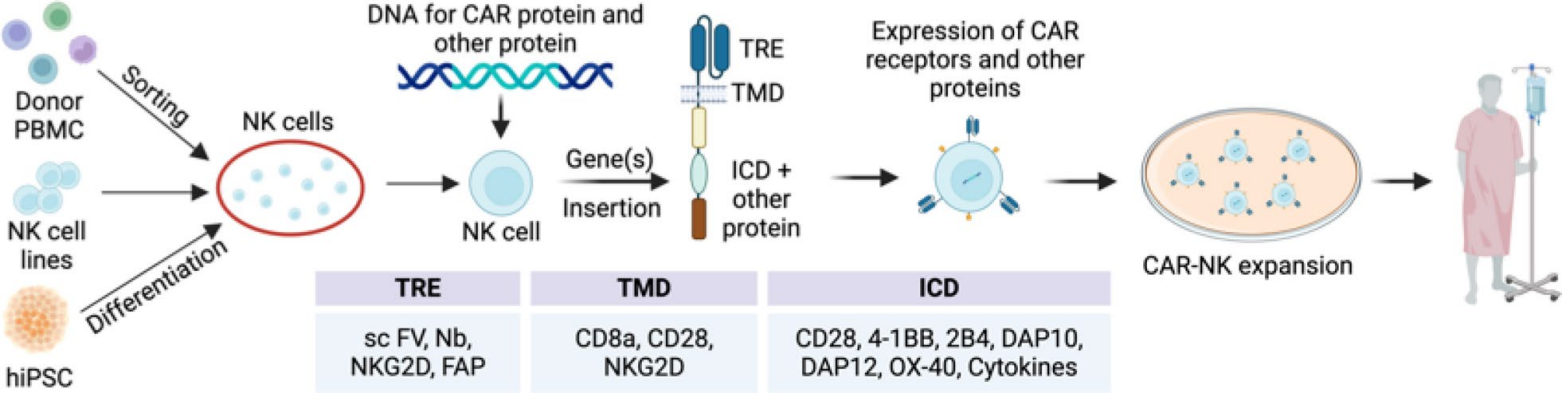
Manufacturing engineered CAR-NK cells for clinical applications.Created with BioRender.com

CAR is a fusion protein which is comprised of four elements expressed and anchored cross the membrane of effector cells through signal peptide-mediated gene delivery (Fig. 3) [28, 29, 72, 73]. A signal peptide (SP), also sometimes referred to as a leader peptide, is a short transient peptide that controls protein secretion and translocation in living cells. In CAR engineering, human CD8α and GM-CSF receptor α chain (GM-CSFRα) are utilized for the SP in six FDA-approved CAR-T products [6, 7].

### Target recognition element (TRE)

Target recognition element in most CARs have utilized a single-chain variable fragment (scFv) derived from a short flexible peptide linker joined together by variable regions of heavy (VH) and light (VL) chains of a conventional monoclonal antibody [74].

CAR cell therapeutic outcomes may decisively be affected by scFv features. scFv’s recognition specificity determines the scope of clinical indications and adverse effects, especially on-target off-tumor toxicity. The ideal target antigen should be exclusive to tumor cells, providing a crucial survival signal for the malignant clone. However, many CAR-T cell targets exhibit shared expression with normal tissues, leading to a certain level of ‘on-target/off-tumor’ toxicity as the target antigen expression patterns are commonplace for the vast majority of target antigens utilized in CAR-T therapies [75–77]. Specific scFv-mediated CAR effector therapies target specific antigen biomarkers on pathogenic tissues, which reflect types and states of diseases [14, 77, 78]. scFv affinity and expression level also play a crucial role in influencing CAR binding affinity, thereby determining the antigen-binding characteristics of the CAR and the efficacy of target cell recognition [79]. Constructing CARs with the precise affinity to discriminate between malignant and normal cells, without inducing any toxicity, is paramount. Numerous studies have illustrated that CARs with reduced affinity can effectively differentiate tumors from normal tissues expressing the same antigen at lower levels. This approach maintains potent antitumor activity and ensures prolonged persistence [80–82]. The scFv design is one of the keys to CAR cell therapeutic success, as a two scFvs strategy with two corresponding scFv target antigens, such as tandem CARs, dual CARs, loop CARs, AND-gate CARs (synNotch-CAR), and inhibitory CARs (iCARs) has been utilized to improve the specificity and affinity of CARs and reduce on-target off- tumor toxicity [77, 83–87]. Boolean-logic gates, such as AND-NOT gates, have employed an inhibitory CAR (iCAR) to selectively suppress CAR-T cell activity at nonmalignant tissue sites. Nevertheless, this strategy appears inefficient, primarily attributed to a kinetic delay in iCAR inhibition of cytotoxicity. Achieving a delicate balance in both CAR and iCAR signaling strength and kinetics is essential to regulate the selectivity of AND-NOT gate CAR-T cells [88].

Most CAR scFvs derived from murine monoclonal antibodies trigger immune response leading to quick clearance of the CAR cells and the risk of disease relapse. Development of humanized scFvs with less immunogenicity may avert the anti-scFv immune response and unnecessary therapeutic failure. Lymphodepleting chemotherapy before CAR cell infusion may significantly reduce the scFv-causing immunogenic response [89–92]. While scFv is commonly employed as the target recognition element in most CARs, it has a notable tendency for self-aggregation. This phenomenon can result in ligand-independent activation, leading to the exhaustion of CAR T cells and subsequently reducing their anti-tumor efficacy [93–95]. The VHH domain, also known as nanobody, represents the smallest antigen-capable fragment with low immunogenicity. It is capable of accessing epitopes that are challenging or impossible to reach with scFvs, making it a promising alternative in CAR cells [96]. The dual anti-BCMA nanobody-based CAR T product, Ciltacabtagene autoleucel (Carvykti®), has received FDA approval [7]. Furthermore, ligands have been incorporated into CAR structures, showing promise in anti-tumor effects and other disease applications. Examples include NKG2D-CAR, FLT3L-CAR, Anti-IL13-zetakine CAR, and CLTX-CAR [16, 97–102]. The antigen-recognition element in the CAR comprises one or more components, which may require a linker to fuse these components. The scFv is a synthetic protein composed of VH and VL chains joined by a linker. The two most common linkers are the GS linker (GGGGS)3 or (G4S)3 and the Whitlow/218 Linker (GSTSGSGKPGSGEGSTKG), with the latter known to enhance scFv affinity. A comparison of G4S and 218 linkers in anti-KDR-CAR T cells revealed no expressional and functional differences between (G4S)5, (G4S)3, and 218 linkers [103]. The selection of the antigen recognition element is pivotal in CAR structure design, exerting a substantial impact on therapeutic specificity, efficacy, and potential adverse effects.

### Hinge domain (HD)

The hinge domain (HD) in CARs functions as a spacer, extending scFvs or other antigen recognition domain beyond the plasma membrane and providing the necessary flexibility to access antigen epitopes on the surface of target cells. Experiments indicate that the efficacy of CARs depends on the hinge domain (HD) length and the distance of the target epitope from the cell membrane. For membrane-distal antigens like CD19, HD incorporation doesn’t enhance killing activity but improves expansion and migratory capacity of anti-CD19 CAR-T cells. When epitopes are closer to the membrane or within glycosylated structures, an appropriate HD length is essential to reduce distance and steric inhibitory effects between the scFv and its epitope [104–106]. The HD derives mostly from IgG1, IgG4, CD8, and CD28 molecules [104]. To minimize potential immunological interactions associated with Ig-based spacers and ensure the safety required for clinical use, spacers derived from components naturally expressed on T cells—such as CD8 and CD28—can be integrated into CAR structures [107]. Irrespective of the scFv origin, CARs with CD8α or CD28 hinge domain/transmembrane domain (HD/TMD) exhibited similar expression levels on the T cell surface and T cell memory phenotype. However, compared to CARs containing the HD/TMD of CD8α, those with CD28 HD/TMD exhibited significantly higher levels of inflammatory cytokines, T cell exhaustion, and activation-induced cell death (AICD) [108].

Removing two consecutive Gly residues in the CD8-derived HD of a second-generation anti-CD19 4-1BB-based CAR was found to reduce spacer flexibility. This modification led to improved tumor control, lower release of inflammatory cytokines in vivo, a downward trend in tumor load, and prolonged survival [109]. Schafer et al. presented a Siglec-derived HD in CAR-T cell to target membrane-proximal target, demonstrating comparable performance to CD8α in a CD20-targeting CAR, with potential cytotoxicity in vitro and in vivo [110]. HD can affect the expression level and functional activity of nanobody-based CARs [111]. HD plays a vital role in optimizing the extracellular structure and ensuring the full functionality of CAR cell therapy.

### Transmembrane domain (TMD)

The TMD serves as a membrane-spanning component that connects the extracellular moiety (antigen recognition domain and hinge domain) to the intracellular signaling domain. It is primarily derived from type-I single-spanning proteins, such as CD4, CD28, and CD8α. The expression level and stability of CAR on T cells were significantly influenced by the TMD rather than the HD. The CD28-derived HD/TMD exhibited a greater tendency for dimer formation compared to CD8α HD/TMD. This dimerization can lead to an increased tonic signal, higher on-target off-tumor toxicity and activation-induced cell death (AICD) [108, 112, 113]. However, CD28 HD/TMD provides a more stable and efficient immune synapse, reducing the antigen-density threshold for T-cell activation in CD19-specific CARs compared to their CD8α counterparts [114].

Optimizing CD8α-derived HD/TMD lengths in the CD19-BBz prototype with co-stimulatory 4-1BB and CD3ζ domains, the Ying group found that CD19-BBz(86) CAR-T cells exhibited a potent and durable antilymphoma response without inducing neurotoxicity or severe cytokine release syndrome (CRS), indicating that modifying the CAR hinge and transmembrane regions can regulate cytokine secretion and contribute to mitigating CAR-T cell-associated toxicities [115]. In a third-generation CAR comprising ICOS and 4-1BB intracellular domains (ICDs), the fusion of the ICOS TMD to the proximal ICOS ICD demonstrated superior antitumor activity and enhanced persistence of CAR-T cells in vivo compared to TMD from CD8α and CD28 [116]. The ICOS TMD enhances interactions between T cells and antigen-presenting target cells [117]. The TMD of 4-1BB in trimeric CARs improves antigen-binding capacity, reducing antigen escape [118]. A KIR-based CAR, utilizing TMD and ICD from KIR2DS2, expressed in DAP12-positive T cells exhibits enhanced in vivo antitumor activity due to increased stability of the KIR/DAP12 complex following antigen engagement [119]. The design of TMD offers opportunities for precise control of CAR receptor functions, helping to insulate it from confounding interactions with endogenous signaling proteins.

### Intracellular domains (ICDs)

#### Co-stimulatory domain

The two-signal model of T-cell activation demonstrates that antigen stimulation without costimulation results in T cell anergy unresponsiveness [120]. The first-generation CARs with antigen recognition domain only exhibited limited antitumor efficacy and poor persistence in vivo [121, 122]. Incorporating one or two costimulatory domains, such as CD28, 4-1BB, CD27, OX40, and ICOS, into the CAR structure significantly enhanced T cell proliferation, persistence, and anti-tumor efficacy in vivo [121, 123, 124]. T cells expressing CD28-costimulated CARs exhibit higher cytokine production but lower persistence, as CD28 does not support human T-cell survival in vivo [116, 125, 126]. Conversely, CARs incorporating the 4-1BB costimulatory domain facilitate long-term survival of T cells in circulation by maintaining a central memory phenotype [127–130]. The persistence of CD28-costimulated CAR-T cells can be improved by replacing CD28 with 4-1BB or CD27 [131–134]. Screening seven different CAR structures with CD28, 4-1BB, and CD80 recombination revealed that the configuration simultaneously utilizing two signaling domains, CD28 and 4-1BB, displayed the highest therapeutic efficacy. This structure demonstrated balanced tumoricidal function, increased T cell persistence, elevated CD8/CD4 ratio, and reduced exhaustion, potentially due to sustained activation of the IRF7/IFNβ and NF-κB signaling pathway by the combined CD28 and 4-1BB [130, 135]. CD28-based CARs chronically activate T-cell exhaustion programs, while 4-1BB-based CARs induce a distinct molecular program and unique T cell differentiation. The 4-1BB-dependent activation of the transcription factor FOXO3 directly contributes to CAR T-cell dysfunction. Costimulatory domains are crucial regulators of CAR-driven T-cell failure, requiring targeted interventions to overcome dysfunction [136]. Additionally, other costimulatory molecules are investigated in CAR structure, including The tumor necrosis factor receptor superfamily TNFRSF5 (CD40), TNFRSF14 (CD270), TNFRSF18 (CD357), Toll-Like Receptor 2 (TLR2) and Dectin-1 [114]. Costimulatory domains can activate diverse downstream signaling pathways. Choosing a costimulatory unit or combination that enhances antitumor activity while ensuring long-term persistence of CAR-T cells is crucial [137].

#### Activation domain

The activation motif is a crucial component in the CAR structure, responsible for triggering T cell activation and functionality. While early CAR development utilized FcγR as the primary activating domain, CD3ζ has become the most common activation molecule in current CAR-T cell designs [4, 104, 114]. All FDA-approved CAR T cell products have employed CD3ζ as the cytoplasmic activation domain [7].

The CD3ζ (CD247) is part of the TCR complex, featuring a small extracellular segment, a transmembrane region, and a long cytoplasmic portion containing three immunoreceptor tyrosine-based activation motifs (ITAMs): ITAM1, ITAM2, and ITAM3. Phosphorylation of these ITAMs recruits the tyrosine-protein kinase ZAP70, initiating downstream signaling cascades. The distal ITAMs (ITAM2 and ITAM3) exhibit lower binding affinity for ZAP-70 compared to ITAM1 [104, 138]. A single functional ITAM is sufficient for potent antitumor efficacy. CARs containing a single ITAM (ITAM1, 2, or 3) outperform triple- and double-ITAM-containing CARs in vivo mouse model and limit T cell differentiation, resulting in more central memory CAR-T cells and prolonged persistence [139]. The CD3ζ module was identified as the source of second-generation CAR tonic signaling across CARs with three different antigen recognition domains. A novel CAR structure was designed using DAP10 exclusively as chimeric costimulatory receptors (CCR), devoid of CD3ζ. These CCR-γδT cells showed no tonic signaling but efficiently activated and exhibited cytotoxic responses in the presence of CCR-specific stimuli or cognate leukemic cells [140].

A KIR-based CAR (KIR-CAR), engineered from the transmembrane and cytoplasmic domains of KIR2DS2 (killer cell immunoglobulin-like receptor 2DS2) without CD3ζ, induces robust antigen-specific proliferation and effector function in vitro when introduced into human T cells with DAP12. T cells modified to express KIR-CAR and DAP12 display superior antitumor activity compared to standard first- and second-generation CD3ζ-based CARs in a xenograft model of mesothelioma highly resistant to immunotherapy [119]. Innovative modifications have been employed to enhance the functionality and safety of CAR cells. Cytokine or cytokine receptor genes integrated into CAR cassettes enhance the activation and persistence of CAR-effector cells through autocrine or membrane-bound mechanisms [141]. The CAR cell-mediated delivery of cytokines (IL-12, IL-7, IL-15, IL-18, and IL-23) [142–147] or other payloads (anti-PD-1 and/or anti-CTLA-4, anti-PD-L1) [148, 149] to the tumor has shown promise in preclinical studies, and some clinical trials have been initiated to further test the efficacy and safety of the fourth or next generation of CAR-T cells [150]. Human CAR-NKTs expressing IL-12 demonstrated potent antitumor activity in leukemia and neuroblastoma tumor models and long-term in vivo persistence [17]. Integration of IL-12 into the CAR exodomain transforms CD8+ T cells into polyfunctional NK-like cells, exhibiting superior killing of antigen-loss tumors [151]. CAR-T cells with local IL-12 release recruit and reinforce macrophage antitumor activity in CAR-T cells-inaccessible tumor lesions [152], executing anti-tumor response in leukemia, ovarian cancer and glioblastoma [153]. CAR-T cells with inducible IL-18 release demonstrated superior activity against large pancreatic and lung tumors [154]. Furthermore, CAR-T cells modified with the 28-ΔIL2RB-z(YXXQ) CAR’s ICD exhibited superior in vivo persistence and antitumor effects in both liquid and solid tumor models. This enhancement was attributed to antigen-dependent activation of the JAK kinase and the STAT3 and STAT5 transcription factors signaling pathways mediated through ΔIL2RB, compared to CAR-T cells expressing a CD28 or 4-1BB co-stimulatory domain alone [155]. The integration of synthetic receptors into the CAR structure enhances the potential of effector cells as powerful therapeutic avenues for the future [147].

#### CAR design for NK cells

The CAR structure was initially introduced into T cells, demonstrating promising therapeutic value along with associated challenges and clinical limitations [24, 156]. Consequently, researchers have extended the use of CARs to other effectors, including NK cells, macrophages, and neutrophils [19, 25, 29, 157–162]. Despite variances in recognition systems between NK and T cells, there exists sufficient similarity that facilitates the utilization of standardized CAR structures formatted with CD3ζ signaling and relevant costimulatory domains as described above.

Antigen-specific 2B4zeta-expressing NK cells engineered by the 2B4 endodomain as costimulatory domain in the CAR structure significantly enhance NK cell immunotherapy of leukemia and other malignancies [163]. In a comparative study of signaling and costimulatory domains in NK-specific molecules, including DAP10 (DNAX-activating protein of 10 kDa), FcεR1γ, CD3ζ, 2B4, and 4-1BB, it was determined that the optimal combinations for CAR-NK cells were 2B4 with CD3ζ and 4-1BB with CD3ζ [164]. Li et al. further designed a CAR construct containing the transmembrane domain of NKG2D, the 2B4 co-stimulatory domain, and the CD3ζ signaling domain to mediate strong antigen-specific NK cell signaling, which was superior to dual CD28-4-11BB costimulatory domains-containing CAR-NK92 cells and induced pluripotent stem cells (iPSC)-derived NK Cells (iPSC-NK cells) [165]. As CAR-NK cells with the CD28 costimulatory domain have demonstrated high efficacy in clinical and preclinical settings [24, 166, 167], it is intriguing to explore whether novel combinations of costimulatory domains could enhance efficacy against tumors. The CD28 homolog CD28H is expressed by NK cells [168]. CD28H-CAR in NK cells triggered lysis of B7H7+ HLA-E+ tumor cells by overriding inhibition by the HLA-E receptor NKG2A. The cytoplasmic domains of CD28H and of the ζ chain were both required for this activity, indicating that CD28H is a powerful activation receptor of NK cells that broadens their antitumor activity and holds promise as a component of NK-based CARs for cancer immunotherapy [169]. While DAP10 as a costimulatory domain in CARs has shown success [170], most experiments indicate that CARs containing DAP10 underperform compared to those with CD28 or 4-1BB [164, 165].

The in vivo persistence of effector cells is crucial for sustained clinical responses. While mature NK cells typically have a short lifespan with limited in vivo persistence in humans [171], persistence of NK cells after transfer require cytokine support. Various cytokines, including IL-2, IL-12, IL-15, IL-18, and IL-21, have been implicated in significantly enhancing NK cell yield and cytotoxic effects against tumor cells. Among these, IL-15 emerges as the most promising cytokine for activating NK cells. Infusion of IL-15 into metastatic malignant patients demonstrated proliferation and expansion of NK cells [172, 173]. The absence of IL-2 or IL-15 may result in a short in vivo lifespan of NK cells [174]. Recent data suggest the presence of a subset of long-lived memory NK cells [175]. Natural killer (NK) cells demonstrate innate memory through brief activation with IL-12 and IL-18, resulting in cytokine-induced memory-like (CIML) NK cell differentiation [176]. Memory-like NK cells (MLNKs) can develop by activating PB NK cells with these cytokines to initiate the memory-like program [19, 177]. In response to target cells, CAR-MLNKs demonstrated markedly increased IFN-γ production and degranulation compared to conventional CAR-NK cells. CD19-CAR MLNKs showed superior persistence and antitumor activity against CD19+ tumors, presenting an appealing approach for treating patients with relapsed or refractory B cell malignancies [178]. CD19-CAR-ML NK cells effectively controlled lymphoma burden in vivo and enhanced survival in human xenograft models [179].

Systemic IL-15 accelerated the ability of responding T cells to kill stimulator-derived memory-like NK cells [180]. The antitumor activity of TRUCKs (T cells redirected for antigen-unrestricted cytokine-initiated killing) involves CAR-induced release of cytokines in the TME [141]. Similar functionality of cytokine-secreting CAR-NK cells is being explored in both tumor and non-tumor diseases. IL-15 “armored” CAR-NK cells are testing clinically [19]. Cord blood NK cells engineered to IL-15-expressiong CD19-CAR demonstrated potent anti-leukemic activity in vitro and improved survival in a murine lymphoma model. IL-15 production critically enhanced their antitumor activity and long-term persistence [23]. Enhancing the antitumor effect, CAR19 NK cells can be fortified with interleukin-15 (IL-15) co-expression to elevate their metabolic fitness and effector function, emphasizing the close connection between the metabolic fitness of CAR-NK cells and their ability to clear tumors [167]. NK cells possess characteristic activation signal pathways, and these should be taken into consideration in CAR structure design to better align with biophysiological features of NK cells, particularly in terms of anti-tumor cytotoxicity.

NK receptor-based domains and cytokine expression offer valuable enhancements to CAR designs, and optimizing combinations of these elements could prove beneficial in clinical applications. Numerous CAR configurations have been evaluated in NK cells, incorporating various combinations of transmembrane regions such as CD16, NKp44, NKp46, and NKG2D and costimulatory domains like 2B4, DAP10, DAP12, or 4-1BB, either alone or in combination with CD3ζ. However, the advantages of integrating NK cell-associated domains compared to conventional domains in CARs have not been systematically investigated [181]. Further refinements are necessary to mitigate potential side-effects associated with cytokine release in clinical settings.

## CAR-NK cell sources and expansion

NK cells inherently possess antitumor capabilities, leveraging their ability to discern a delicate equilibrium between activating and inhibitory ligands expressed on tumor cells [182]. The intricate mechanisms that govern the immune surveillance of tumors, in the absence of prior antigen sensitization, warrant further comprehensive characterization. NK cell anti-tumor potential has been currently enhanced by different therapeutic approaches, including NK cell activation with cytokines, blockade of NK cell inhibitory receptors or inhibitory pathways with antibodies or inhibitors, NK cell engagement to tumors via the multi-specific molecules and CAR-engineered NK cells [182]. NK cells armed with the next generation of CAR multi-functional structural modifications are poised to offer expansive clinical prospects.

### NK cell lines

NK cell lines such as NK-92 cells have been utilized for the initial study of CAR-NK cells. NK cell lines have superior robustness and proliferative capacity with a long lifespan property to gene engineering. NK-92 cells without CD16 expression do not mediate ADCC-based cell killing [29]. NK-92-derived cell products need irradiation prior to patient administration, which can negatively affect in vivo persistence and therapeutic potential [24]. CAR-NK-92 cells based on CD28-CD3ζ signaling domain have anti-tumor cytotoxicity targeting EpCAM and ErB2 breast cancer cells, EGFR on glioblastoma cells and breast cancer brain metastases, CD19 on B-cell malignancies, CS1 on multiple myeloma cells, and CD33 on acute myeloid leukemia cells [183]. CAR-NK-92 cells are based on 4-1BB- CD3ζ signaling domain for targeting ErbB2, CD19 and EBNA3C. CAR-NK-92 cells based on CD28-4-1BB-CD3ζ target Wilms tumor protein with HLA-A2 complex for the elimination of CD3 or CD5 expressing malignant T cells [183]. Evidence suggests the CARs in NK-92 could link to endogenous signaling pathways of NK cell cytotoxicity to enhance antitumor effects [184].

### PB-NK and CB-NK

NK cells can be harvested from peripheral blood (PB-NK cells) or from umbilical cord blood (CB-NK cells) [29], with PB and CB offering attractive, allogeneic, off-the-shelf sources of NK cells for CAR-NK cell immunotherapy [23, 185]. CB-derived NK cells show an immature phenotype expressing a relatively higher percentage of inhibitory receptors (CD94/NKG2A and KIRs) and less adhesion molecules, and the limited volume of an CB unit make it a major challenge to obtain sufficient numbers of NK cells as off-the-shelf bank for clinical application [186].

Different PB-NK subsets, such as NKG2A−KIR−, NKG2A+KIR−, NKG2A+KIR+, and NKG2A−KIR+NK cell subsets, that have been transduced with the retroviral CD19-CAR vector show stable similar expression levels of CAR, which enhance in vitro NK cytotoxicity against CD19+ tumor cell lines. C19-CAR PB-NK cells retain the expression and function of their native activating receptors as their in vitro killing activity does not require the engagement of activating NK receptors. The antileukemia activity of CD19-CAR PB-NK cells is superior to that of CAR-T cells the in vivo experimental models [187]. Clinical application of CAR PB-NK cells necessitates a robust manufacturing process, as demonstrated by Quintarelli et al.’s development of a feeder-free, fetal bovine serum (FBS)-free approach that yielded a substantial 13,000-fold expansion over a 30-day period and did not reach a plateau at the culmination of the culture. The feasibility of manufacturing an “off-the-shelf” CAR PB-NK cell bank may benefit many different recipients [187]. As aforementioned, MLNKs can be prepared from PB-NK cells through stimulation with specific cytokines [19, 177, 188]. CAR-MLNKs show enhanced responses against resistant cancers with more flexible targets compared to conventional NK cells [179]. While CAR-NK cells only are present for several months [189], mbIL-15 modified CAR-MLNKs may further enhance in vivo persistence and antileukemia activity [176].

### iPSC-NK cells

iPSC-NK cells offer precise genome editing prior to NK cell differentiation process. iPSCs can grow indefinitely in an undifferentiated state via self-renewal for generation of an unlimited number of uniform NK cells [190]. Numerous genetic alternations have been intentionally engineered to enhance the biology and function of iPSC-derived NK cells with enhanced expansion, in vivo persistence and tumor killing capability for therapeutics [190, 191]. iPSC-derived iDuo NK cells were engineered with three components of CD19-CAR, non-cleavable CD16 (hnCD16) and a membrane-bound IL-15/IL-15R (IL-15RF) fusion protein to effectively eliminate both CD19 and CD20 lymphoma under anti-CD20 mAb and to extend their in vivo persistence [192]. A clinic trial using FT536 (hnCD16/CD38KO/anti-MICA/B CAR/IL-15RF) iPSC-derived NK cells with monoclonal antibodies demonstrate the safety and early indication of efficacy from the interim clinical results [191].

## Generation of CAR-NK cells

### Viral delivery

Compared to the production of CAR-T cells, the generation of CAR-NK cells poses greater challenges due to NK cell nature of a shorter lifespan, difficulties in activation and expansion, and reduced efficiency in lentiviral transduction with current protocols [193]. CAR constructs can be introduced into NK cells through either viral delivery or non-viral delivery methods. Retroviral and lentiviral vector systems are frequently employed to efficiently transduce NK cells with CAR-containing viral particles. Pre-stimulating human NK cells with human IL-2 (500 U/mL) for a period of 1–5 days led to a significant increase in GFP + NK cells compared to immediate transduction after isolation. High viability was observed for over 3 days with pre-stimulation using the VSVG-pseudotyped pLV-mPGK-GFP vector system [194]. A strategy involving pre-transduction incubation with irradiated lymphoblastoid (LCL) feeder cells plus IL-2, along with the TBK1 inhibitor BX795, resulted in efficient lentiviral integration (mean of 23% transgene+ NK cells) and successful subsequent proliferation of the transduced cells. Among eight promoter candidates, the short human EF1α promoter demonstrated the best performance for bicistronic CAR expression in primary NK cells [194]. Recently, the myeloproliferative sarcoma virus (MPSV) promoter has emerged as particularly noteworthy, exhibiting the highest level of transgene expression when compared to several other promoters including human short and optimized EF1α, hPGK, and the two enhanced SFFV promoters [166].

In research settings and clinical trials, cationic culture additives such as polybrene and Vectofusin, as well as human fibronectin derivatives like CH-296 or Retronectin, have been employed to enhance the binding of lentiviral particles to the surface of target cells. This aims to increase the likelihood of viral entry, thereby improving the efficiency of genetic modification for both adherent and non-adherent cells, including primary NK cells. However, a systematic comparison of these reagents has not been conducted yet [166]. Colamartino et al. found that Baboon envelope pseudotyped lentiviral vectors (BaEV-LVs) demonstrated superior performance when compared to Vesicular Stomatitis Virus type-G (VSV-G), RD114, and Measles Virus (MV) pseudotyped LVs [195]. The cellular glycoproteins (ASCT-1 and ASCT-2) acting as viral receptors exhibited higher expression levels on activated NK cells as opposed to naïve or resting NK cells. Notably, IL-2 priming of human NK cells induces the upregulation of the glutamine transporter ASCT2, thereby facilitating lentiviral infection [166, 195–197].

### Nonviral delivery

Nonviral gene transfer technologies are being developed to replace viral vectors for CAR cells delivery. Transposon-based gene-delivery systems have been developed and refined for over 20 years. Transposon systems have been extensively utilized for delivering CARs to T cells in the treatment of both hematological and solid tumors [198]. Early-phase clinical studies using transposon system (the Sleeping Beauty or PiggyBac system) have demonstrated efficacy and safety with no evidence of malignant transformation or integration into known oncogenes [199–201]. CAR-NK cells generated through the TcBuster transposon system exhibit enhanced antigen-specific antitumor activity in vitro [202].

CAR mRNA was delivered to > 70% of cultured T cells with a 7-day expression period by biodegradable nanocarriers. When administered periodically, CAR- -encoding mRNA particles can genetically reprogram circulating T cells to induce antitumor responses with similar efficacies compared to conventional adoptively transferred T cells that have been virally transduced ex vivo [14, 203]. The feasibility of mRNA electroporation for CAR engineering of short-term activation NK cells has been demonstrated. CAR-NK cells delivered via mRNA retain their sensitivity to innate signals and exhibit varying cytotoxicity profiles dictated by their subsets [204]. mRNA transfection through electroporation or lipid nanoparticles leads to robust but transient CAR expression.

### CAR-NK cells expansion

A vast number of CAR-NK cells are required for clinical therapeutics, a dose range of 10^8^–10^10^ cells per patient used in a CAR-NK clinical trial. The feeder cell culture system is regarded as the most effective system to achieve clinical doses of CAR-engineered primary NK cells, which use immortalized cells lines as artificial antigen-presenting cells and expressed with membrane-bound (mb) stimulatory molecules (mbIL-15, mbIL-21, and 4-1BBL) [23, 24, 205–207]. The choice of feeders with distinct stimulatory molecules has a notable impact on NK functionality and the degree of expansion. An approach involving irradiated mbIL-21/4-1BBL-expressing K562 feeder cells has resulted in a remarkable near-50,000fold expansion of CAR-NK cells within a span of 21 days [205, 208, 209]. The use of autologous feeder cells such as irradiated PBMCs has demonstrated success in expanding NK cells to clinical-dose numbers [210].

To mitigate potential risks associated with feeder cells, feeder cell-free systems for CAR-NK cell expansion are under development. These systems utilize high doses of stimulating cytokines coated or non-coated on materials. However, they have yet to surpass the degree of CAR-NK cell expansion achieved with feeder cell-based methods [211–216]. Additionally, the supplementation of the medium with human AB serum is often necessary for clinical use, primarily to address concerns related to xenosensitization and non-specific inflammation induced by fetal calf serum. Serum-defined or serum-free supplements are now emerging in the market, demonstrating comparable performance to human AB serum [217]. Clinical CAR cell manufacturing can be performed manually in a GMP-certified lab or by using an automated closed system [218]. Point of care manufacturing of CAR-T cells on the automated CliniMACS Prodigy® device allows reproducible and fast delivery of cells for the treatment of patients [219]. A standardized GMP-compliant overall process has been optimized by integrating the clinical-scale expansion procedure into the automated and closed Prodigy system. This integration encompasses in-process control (IPC) samples, quality controls, and optimal time frames for NK cell transduction with CAR vectors [220].

## Preclinical studies of CAR-NK

CD19 CAR-NK cells from NK-92, PB, CB and iPSC showed cytotoxicity against B-cell malignancies in vitro and in vivo [23, 174, 187]. CAR-NK cells have the potential to eradicate tumor cells with little or no expression of the Car-target through the innate cytotoxicity and ADCC properties inherent to NK cells [221]. CD19 CAR-NK cells with ectopic IL-15 secretion have been shown to exhibit a comparable kinetic of in vitro expansion and enhanced antitumor activity in vivo compared to CD19 CAR-NK cells without IL-15. IL-15 plays a crucial role in augmenting the proliferation, persistence, and homing of CD19 CAR cord blood-derived NK cells in a lymphoma-xenograft mouse model [23]. In an in vivo animal model utilizing a CAR structure with a second-generation intracellular domain (4-1BB-CD3ζ), the data demonstrated that the antileukemia activity of CAR.CD19-NK cells is superimposable to that of CAR-T cells with a lower xenograft toxicity profile [187]. Anti-BCMA CAR-NK cells with CXCR4 mRNA electroporation reduced significantly multiple myeloma (MM) tumor burden and extended the survival of the tumor-bearing mice [206]. Alternative CAR targets were explored in the treatment of hematological malignancies such as CD3, CD5, CD7, CD33, CD138, SLAMF7 and NKG2D ligands in pursuit of for more effective strategies [174]. Additionally, the nanobody-based BCMA CAR-NK exhibited remarkable specific killing ability in vitro, BCMA-CD28-IL15 CAR-NK especially inhibited the growth of tumor cells and prolonged survival of MM mouse model [222]. The nanobody-based CD5 CAR-NK cells demonstrated greater antitumor activity in T-cell malignancies. Nanobody-based CAR-NK immunotherapy has been exploited successfully [223].

In the realm of solid tumors, CAR-T cell therapies are at an early stage of development, encountering limitations in efficacy attributed to the intricate mechanisms of tumor escape and the challenge of a solid TME [20]. Similarly, the advancement of living drugs through CAR-NK cell-based approaches is currently in the exploratory phase, predominantly within the context of preclinical investigations. HER2 CAR-NK-92 cells manifested specific antitumor cytotoxicity against ErbB2-expressing breast cancer cells in vitro. Additionally, they significantly reduced metastasis formation of ErbB2-expressing renal cell carcinoma cells [184].

EGFR-CAR-NK cells demonstrated a substantial inhibition of tumor growth in mouse models derived from triple-negative breast cancer (TNBC) cell lines (CLDX) and patient-derived xenografts (PDX) [224]. Reports have documented the utilization of CAR-NK cells targeting various antigens, including HER2, EGFRvIII, IL-13Rα2 in glioblastoma, and HLA-G, CD24, CD44, CD133, Mesothelin, and folate receptor alpha (αFR) in ovarian cancer [174]. Given that IL-15 activates and enhances the survival of CD8 T cells and NK cells, anti-prostate stem cell antigen (PSCA) CAR-NK cells engineered with a soluble IL-15 have demonstrated the capability to suppress tumor progression and prolong survival in a mouse model of metastatic pancreatic cancer [225].

Primary NK cells and NK-92MI cell line engineered with CD147-CAR molecules can specifically kill malignant HCC cell lines in vitro and effectively control progression of HCC mouse models. CD147-synNotch-inducible GPC3-CAR-NK cells can eliminate CD147+ GPC3high HepG2 cells [226]. Among the four types of c-Met CAR-NK-92 cells transduced with CAR structures of the same NKG2D and CD3ζ as transmembrane and activating domains but with four costimulatory domain combinations (4-1BB, 2B4, 4-1BB-2B4, 2B4-DAP10), c-Met CAR-NK-92 cells with the intracellular domain of 2B4-DAP10-CD3ζ exhibited superior cytotoxicity in vitro in a concentration-dependent manner for high c-Met expression LUAD (lung adenocarcinoma) cell lines and inhibited non-small cell lung cancer (NSCLC) xenograft growth in vivo [227].

## Clinical studies of CAR-NK cell therapy products

While promising results from preclinical studies involving CAR-NK cells are present in the literature, there have been relatively few reports regarding the clinical application of CAR-NK cells.

Notably, Reznavi’s group has described pioneering clinical studies. In such studies, HLA-mismatched anti-CD19 CAR-NK cells were derived from CB-NK and engineered to express an anti-CD19 CAR, interleukin-15, and an inducible caspase 9 safety switch for ex vivo expansion. In a study involving 11 patients, these cells were administered in a single infusion at 1 × 10^5^–1 × 10^7^ CAR-NK cells per kilogram of body weight after lymphodepleting chemotherapy. The results showed a response rate of 73%, with 63.6% achieving complete remission (CR). Notably, none of the patients experienced cytokine release syndrome, neurotoxicity, or graft-versus-host disease. Additionally, there was no significant increase in the levels of inflammatory cytokines, including interleukin-6 [24]. Trogocytosis was identified as the underlying mechanism for relapse following CAR-NK cell therapy by causing tumor antigen loss and NK cell exhaustion due to fratricide. A new strategy was developed to offset the trogocytosis-mediated mechanism by a dual-CAR system comprising of an activating CAR against the cognate tumor antigen and an inhibitory CAR recognizing NK-self molecule, leading to enhanced CAR-NK antitumor cytotoxicity [228]. Following the engineering of NK cells with an RNA electroporation approach of NKG2D-DAP12 CAR, clear therapeutic benefits against tumors were observed in both in vitro and in vivo settings. In a clinical context, three patients with metastatic colorectal cancer were treated with local infusion of the CAR-NK cells. The results demonstrated a marked decrease in tumor cells in the ascites of two patients and rapid tumor regression in the metastatic liver of another patient. These findings underscore the promising therapeutic potential of utilizing RNA CAR-NK cells in advanced colorectal cancer [207]. In a case study, anti-ROBO1 CAR-NK-92 cells were administered to an individual with pancreatic cancer and liver metastases through systemic infusions and intratumoral injections, resulting in stable disease for 5 months, with fever being the only reported adverse event [229]. In a recent major clinical study on allogeneic CD19-specific CAR-NK cells targeting CD19+ B cell tumors, the Rezvani group reported 1-year overall survival and progression-free survival rates of 68% and 32%, respectively. Notably, the study observed no significant toxicities, including cytokine release syndrome, neurotoxicity, or graft-versus-host disease [230]. CRS is a symptomatic disorder caused by on-target tissue damage due to increased levels of proinflammatory cytokines from large numbers of immune cells. The decreased prevalence of CRS associated with CAR-NK therapy may be related to the nature of NK innate immune cells. As opposed to T cells, NK cells have a characteristically more-controlled-less-aggressive response and different landscape of released cytokines, possibly due to decreased interleukin-6 production and different cross-talk with myeloid cells [11].”

There are an increasing number of clinical trials involving targets such as CD5, CD7, CD19, CD20, CD22, CD33, CD38, CD70, HER2, Mesothelin, Muc1, NKG2D ligands, PD-1, PD-L1, PSMA, ROBO1, CD19/CD22, CD33/CLL1, CD38/SLAMF7, NKG2D(NKG2D-CD8-DAP12-CAR) (NCT03415100), FT536 (hnCD16/CD38KO/anti-MICA/B CAR/IL-15RF), for hematological malignancies and solid tumors [29, 174, 191]. New clinical trials involving CAR-NK cells are on the rise globally, including Dual CAR-NK19/70 trials for R/R B-cell lymphomas and advanced solid tumors (NCT05842707 and NCT05703854), TROP2-CAR-NK (TROP2-CAR engineered IL15-transduced CB-NK cells) trials (NCT06066424 and NCT05922930) for advanced solid cancers, NKG2D CAR-NK trial for ovarian cancer (NCT05776355), and Anti-CD19 CAR-NK (KN5501) trial for Systemic Lupus Erythematosus (SLE) (NCT06010472). The clinical trials for CAR-NK cell therapy within the last 2 years can be seen in Table 1. The insights gained from these clinical trials will significantly contribute to our understanding of the efficacy and safety of CAR-NK cell therapy. Moreover, they will serve as instructive references for the design of new CAR-NK therapies. Overall, a significant advantage of CAR-NK cell therapy observed in ongoing clinical trials is its excellent safety profile. This includes a reduced risk of neurotoxicity or cytokine release syndrome (CRS), along with the potential for using allogeneic cells [181].

**Table 1:** Clinical Trials for CAR-NK cells (2022–2024)

Antigen target	Disease target	Interventions	NCT Number	Sponsor	First Posted	Locations	Study Status	Data
BCMA	MM|PCL	Human BCMA targeted CAR-NK cells injection	NCT06045091	Hrain Biotechnology Co., Ltd	2023	China	RECRUITING	Not yet
BCMA	MM	BCMA CAR-NK	NCT05652530	Shenzhen Pregene Biopharma Co., Ltd	2022	China	RECRUITING	Not yet
BCMA	MM	FT576 (Allogenic CAR NK cells with BCMA expression)	NCT05182073	Fate Therapeutics	2024	USA	NOT_YET_RECRUITING	Not yet
CD123	R/R AML	CD123-CAR-NK cells	NCT05574608	Affiliated Hospital to Academy of Military Medical Sciences	2022	China	RECRUITING	Not yet
CD123	R/R AML	JD123 injection	NCT06201247	Peking University People’s Hospital	2024	China	RECRUITING	Not yet
CD123	AML|Blastic Plasmacytoid Dendritic Cell Neoplasm (BPDCN)|Relapse Leukemia|Refractory Leukemia	CD123 targeted CAR-NK cells	NCT06006403	Chongqing Precision Biotech Co., Ltd	2023	China	RECRUITING	Not yet
CD19	ALL	CAR-NK-CD19 Cells	NCT05563545	Shanghai Simnova Biotechnology Co.,Ltd	2022	China	COMPLETED	Not yet
CD19	ALL|B-cell Lymphoma|CLL	allogenic CD19-CAR-NK cells	NCT05739227	Xuzhou Medical University	2023	China	RECRUITING	Not yet
CD19	Diffuse Large B Cell Lymphoma	anti-CD19 CAR NK cells	NCT05673447	Changhai Hospital	2023	China	RECRUITING	Not yet
CD19	B-Cell Lymphoblastic Leukemia/Lymphoma	Anti-CD19 UCAR-NK cells	NCT05654038	920th Hospital of Joint Logistics Support Force of People’s Liberation Army of China	2022	China	RECRUITING	Not yet
CD19	SLE	anti-CD19 CAR NK cells (KN5501)	NCT06010472	Changhai Hospital	2023	China	RECRUITING	Not yet
CD19	B-cell Lymphoma|B-cell Leukemia	anti-CD19 UCAR-NK cells	NCT05570188	Kunming Hope of Health Hospital	2022	China	WITHDRAWN	Not yet
CD19	B-cell NHL	anti-CD19 CAR-NK	NCT05472558	Second Affiliated Hospital, School of Medicine, Zhejiang University	2022	China	RECRUITING	Not yet
CD19	ALL, CLL, NHL	CAR-NK-CD19 Cells	NCT05410041	Beijing Boren Hospital	2022	China	RECRUITING	Not yet
CD19	R/R B-cell Hematologic Malignancies (Adult)	CD19-CAR-NK	NCT05645601	Affiliated Hospital to Academy of Military Medical Sciences	2022	China	RECRUITING	Not yet
CD19	B-ALL(Repapse)	CD19 CAR-NK	NCT06631040	Shahid Beheshti University of Medical Sciences	2024	Iran	NOT_YET_RECRUITING	Not yet
CD19	B-Cancer	NKX019 (allogeneic CAR-NK)	NCT05020678	Nkarta, Inc	2024	USA	Recruiting	Not yet
CD19	NHL (R/R)	TAK-007	NCT05020015	Takeda	2024	USA	NOT_YET_RECRUITING	Not yet
CD19	NHL (R/R)	CD19CAR NK	NCT06206902	Shanghai Simnova Biotechnology Co.,Ltd	2024	China	Recruiting	Not yet
CD19	Refractory B Cell-mediated Autoimmune Diseases	CNTY-101 (a CD19-targeted CAR iNK Cel	NCT06255028	Century Therapeutics, Inc	2024	USA	Recruiting	Not yet
CD19	NHL (R/R)	CARCIK-CD19	NCT05869279	Fondazione Matilde Tettamanti Menotti De Marchi Onlus	2024	Italy	Recruiting	Not yet
CD19 (high affinity CD16, cyclophosphamide, fludarabine, Rituximab)	R/R NHL	A CD19t-haNK suspension,Cyclophosphamide, Fludarabine|DRUG: Rituximab	NCT05618925	ImmunityBio, Inc	2022	USA	NOT_YET_RECRUITING	Not yet
CD19 (IL-2 and lymphodepleting Chemotherapy)	R/R CD19-Positive B-Cell Malignancies|Indolent NHL|Aggressive NHL	CNTY-101, IL-2, Lymphodepleting Chemotherapy	NCT05336409	Century Therapeutics, Inc	2022	USA	RECRUITING	Not yet
CD19/CD70	B-cell NHL	CB dualCAR-NK19/70	NCT05667155	Second Affiliated Hospital, School of Medicine, Zhejiang University	2022	China	RECRUITING	Not yet
CD19/CD70	R/R B-cell NHL	dualCAR-NK19/70 cell	NCT05842707	Aibin Liang, MD, Ph.D	2023	China	RECRUITING	Not yet
CD33/CLL1 (Cyclophosphamide/Fludarabine/Ctarabine	AML, Adult|Minimal Residual Disease	CD33/CLL1 dual CAR-NK cell|DRUG: Cyclophosphamid|DRUG: Fludarabine|DRUG: Cytarabine|DRUG: CD33 CAR-NK cell|DRUG: super NK cell	NCT05987696	Institute of Hematology & Blood Diseases Hospital, China	2023	China	NOT_YET_RECRUITING	Not yet
CD5 (IL-15 transduction,Fludarabine, Cyclophosphamide))	Hematological Malignancy	Fludarabine Phosphate, Cyclophosphamide, CAR.5/IL15-transduced CB-NK cells	NCT05110742	M.D. Anderson Cancer Center	2021	USA	NOT_YET_RECRUITING	Not yet
CD70 (IL-15 transduction, Fludarabine, Cyclophosphamide)	Advanced Renal Cell Carcinoma|Advanced Mesothelioma|Advanced Osteosarcoma	CAR.70/IL15-transduced CB-derived NK cells, Fludarabine phosphate, Cyclophosphamide	NCT05703854	M.D. Anderson Cancer Center	2023	USA	RECRUITING	Not yet
CD70 (IL-15 transduction, Fludarabine)	B-Cell Lymphoma|MDS|AML	CyclophosphamidE, CAR.70/IL15-transduced CB-NK cells, Fludarabine phosphate	NCT05092451	M.D. Anderson Cancer Center	2021	USA	RECRUITING	Not yet
Claudin6, GPC3, Mesothelin, or AXL	Stage IV Ovarian Cancer|Testis Cancer, Refractory|Endometrial Cancer Recurrent|CAR NK	Claudin6, GPC3, Mesothelin, or AXL targeting CAR-NK cells	NCT05410717	Second Affiliated Hospital of Guangzhou Medical University	2022	China	RECRUITING	Not yet
CLL1	AML, Adult	CLL1 CAR-NK cell injection	NCT06027853	Zhejiang University	2023	China	RECRUITING	Not yet
CLL1 or CD33	AML (Adult)	iPSC -CAR-NK	NCT06367673	The first affiliated hospital, Zhejiang University	2024	China	Recruiting	Not yet
DLL3	SCLC, Extensive Stage	DLL3-CAR-NK cells	NCT05507593	Tianjin Medical University Cancer Institute and Hospital	2022	China	RECRUITING	Not yet
NKG2D ligands	Ovarian Cancer	NKG2D CAR-NK	NCT05776355	Hangzhou Cheetah Cell Therapeutics Co., Ltd	2023	China	RECRUITING	Not yet
NKG2D ligands	AML	NKG2D CAR-NK	NCT05734898	Zhejiang University	2023	China	RECRUITING	Not yet
NKG2D ligands	AML, MDS	NKX101-CAR NK	NCT04623944	Nkarta, Inc	2024	USA	NOT_YET_RECRUITING	Not yet
SZ003	HCC (Advanced)	A SZ003 CAR-NK	NCT05845502	Shantou University Medical College	2023		NOT_YET_RECRUITING	Not yet
SZ011	Ovarian Epithelial Carcinoma	SZ011 CAR-NK	NCT05856643	Shantou University Medical College	2023		NOT_YET_RECRUITING	Not yet
SZ011	Advanced Triple Negative Breast Cancer	SZ011 CAR-NK	NCT05686720	First Affiliated Hospital of Shantou University Medical College	2023	China	NOT_YET_RECRUITING	Not yet
TROP2	Colorectal Cancer (CRC) With Minimal Residual Disease (MRD)	TROP2-CAR-NK	NCT06358430	M.D. Anderson Cancer Center	2024	USA	NOT_YET_RECRUITING	Not yet
TROP2 (Cyclophosphamide and Fludarabine)	Pancreatic Cancer|Ovarian Cancer|Adenocarcinoma	TROP2-CAR-NK|DRUG: Cyclophosphamide|DRUG: Fludarabine	NCT05922930	M.D. Anderson Cancer Center	2023	USA	RECRUITING	Not yet
TROP2 (Cyclophosphamide and Fludarabine)	Solid Tumors	Rimiducid, TROP2-CAR-NK Cells, Fludarabine phosphate, Cyclophosphamide	NCT06066424	M.D. Anderson Cancer Center	2023	USA	RECRUITING	Not yet

## Advantages and limitations of CAR-NK cell therapy

The advantages of CAR-NK cell therapy are evident, capitalizing on the intrinsic natural cytotoxicity of NK cells against pathogenic cells. Additionally, there is a further enhancement of specificity and functionality through CAR-mediated mechanisms, complemented by relevant gene modulation and genomic editing [15]. CAR-NK cells often exhibit flexibility in targeting tumor associated antigens, making them applicable to a variety of cancers. CAR-NK cells represent "off-the-shelf" products that can be derived from allogeneic sources PB, CB, and PSCs without the need for HLA match between the donor and recipient. The development of CAR MLNKs enhances persistence and long-term efficacy of antitumor responses in vivo. NK cells are known for their innate recognition and killing of pathogenic cells without requiring prior sensitization, reducing the risk of off-target effects. CAR-NK therapy may have a lower incidence and severity of CRS and neurotoxicity and much less risk of GvHD [15].

The definitive efficacy and safety of CAR-NK cell therapy are contingent upon the identification of precise tumor neoantigens [231]. The lack of tumor-specific antigens (TSAs) poses a substantial obstacle to the implementation of CAR-NK cell therapy across a spectrum of diseases. Although cytokine-induced memory-like NK cells contribute to extended in vivo persistence, the comprehensive realization of the therapeutic efficacy of CAR-NK cells faces notable challenges. These challenges encompass issues associated with tumor antigen loss or escape, fratricide and exhaustion of CAR-NK cells. Furthermore, intricacies related to the penetration and trafficking within TME, coupled with considerations of fitness and immune suppression within the TME, add to the complexity [30]. This constellation of factors may necessitate recurrent infusions of CAR-NK cells to achieve sustained efficacy.

Meanwhile, the development of standardized and cost-effective manufacturing processes for large-scale clinical CAR-NK cell products faces significant challenges. Despite promising outcomes in preclinical and early clinical studies, the overarching clinical experience with CAR-NK therapy is in a state of evolution. Further comprehensive studies are imperative to establish the long-term safety and efficacy of novel CAR-NK cell therapeutics. Therefore, comprehending these advantages and limitations is pivotal for the continual refinement and optimization of CAR-NK therapies, aiming for enhanced outcomes in the realm of cancer treatment and beyond. While both CAR-NK and CAR-NKT cells share common features in targeting and killing cancer cells, they originate from different immune cell types and possess distinct biological functions, influencing their therapeutic potential and application [162]. CAR-NK cells exhibit distinct characteristics when compared to both CAR-T cells and traditional NK cells, as summarized in Table 2. Although autologous CAR T cells outperformed allogeneic CAR NK cells in CAR-mediated antitumor effector functions in vitro and in vivo [232], CAR-based cell therapy is evolving beyond the initial focus on CAR-T cells to include CAR-NK cells and CAR macrophages [233]. A comparative analysis of the latest developments in these approaches is presented in Table 3.

**Table 2:** CAR-NK cell features

Parameter	Parameters	NK cells Modification or Characteristication
CAR-NK CAR structure	TRE	scFv or Nanobody (mono-, bi- or mult-valent), NKG2D, FAP
	HD/TMD	CD4, CD8a, CD28, NKG2D
	ICD	CD28, 4-1BB, 2B4, DAP10, DAP12, OX-40, CYTOKINES
	Innate target recognition	Like conventional NK cells, CAR-NK cells retain their innate ability to recognize and target cells without prior sensitization
CAR-NK properties	Versatility	Can be derived from various sources, including PBMC, CB, iPSC or cell lines
	Allogeneic potential	Allogeneic CAR-NK cells from healthy donors may offer an off-the-shelf therapeutic option
	Enhanced cytotoxicity	CAR-NK cells exhibit improved cytotoxicity against target cells upon antigen binding
	GvHD	Allogeneic CAR-NK cells may have a lower risk of GvHD compared to CAR-T cells
CAR-NK cells vs. CAR-T cells	Source	CAR-NK cells can be derived from various sources, offering potential allogeneic options. CAR-T cells are typically autologous
	Persistence	CAR-T cells tend to persist longer in the body compared to CAR-NK cells
	Immunogenicity	CAR-NK cells may be less immunogenic than CAR-T cells, reducing the risk of adverse immune reactions
CAR-NK cells vs. conventional NK cells	Target specificity	CAR-NK cells are engineered for specific antigen recognition, whereas conventional NK cells rely on a broader range of activating and inhibitory receptors
	Cytotoxicity	CAR-NK cells exhibit enhanced cytotoxicity against target cells compared to unmodified NK cells
	Versatility	CAR-NK cells can be customized for various cancer types, potentially increasing their versatility

**Table 3:** Comparison of CAR-T cells, CAR-NK cells, and CAR-macrophages in immunotherapy

Parameter	CAR-T cells	CAR-NK cells	CAR macrophage
Cell source	Autologous or MHC-matched allogeneic T cells	Autologous, non-MHC-matched allogeneic NK cells or NK cell lines. Rich cell source	Autologous. Preclinical studies use iPSCs-derived macrophage and macrophage cell lines
HD/MD	CD4, CD8a, CD28	Similar to CAR-T structure, but can use NK-specific HD/TMD	Similar to CAR-T structure, but could use macrophage-specific HD/TMD
ICD	CD3ζ plus a costimulatory domain, CD28, 4-1BB and others	Similar to the CAR-T structure, it can utilize NK-specific signaling domains like 2B4, DAP10, and DAP12	Similar to the CAR-T structure, this design allows for the incorporation of alternative ITAM-containing signaling domains. Additional ligands can be employed, not for triggering phagocytosis, but rather to modify the tumor microenvironment
CAR transduction	Primary T cells	Primary cells, iPSCs or cell lines	Primary cells, iPSCs or cell lines
In vitro expansion	Yes	Yes for autologous NK cells. Cell line can be expanded after transduction and selection	Yes for autologous macrophages. iPSC and cell lines can beexpanded after transduction and selection
Cytotoxicity mechanisms	CAR-dependent cell	Both CAR-dependent and CAR independent NK-cell natural cytotoxicity	CAR-dependent phagocytosis by macrophages, macrophage-driven immunostimulatory TIME, macrophage-induced tumor microenvironment alterations, and macrophages acting as antigenpresenting cells for immune response stimulation
Cytokine release syndrome and neurotoxicity	Common and often serious	Less common and less serious	No clinical data
Infiltration into TME	Typicall scare	Moderately common	Generally abundant
Clinical experience or trial	Established effectiveness, with five FDA-approved CART therapies	o FDA-approved NK cell therapies yet. However, at least one trial has been published demonstrating a superior safety profile	No approved therapies, and limited clinical data is available
Off-the-shelf CAR product	Unlikely. Usually autologous or MHC-matched allogeneic CAR-T cells	Yes with NK cell lines; potential with allogeneic NK cells, but poor recovery when cryopreserved	Theoretically possible with macrophage cell lines. However, there is no available clinical data
Persistence	Can persist in the body, providing sustained effects	Typically have a shorter lifespan in the body. Memory-like NK cells	Their lifespan varies, and they can be influenced by the tissue
Immunogenicity	Potential for cytokine release syndrome and neurotoxicity	Generally considered less immunogenic compared to CAR-T cells	Can modulate immune responses but may have limitations in terms of systemic use
Clinical applicability	Primarily used in hematological malignancies	Maybe various cancers, especially in the context of allogeneic therapy. Maybe infectious disease	Different applicability

Moreover, CAR γδ T cells stand out for their ability to demonstrate features of both the innate and adaptive immune response, enabling antitumor activity that is MHC-independent. Recent preclinical studies have demonstrated the effectiveness of CAR-γδ T cell cytotoxicity against hematologic malignancies [234]. A variety of strategies for engineering γδ T cells have emerged, unveiling considerable therapeutic potential [235]. Anti-CD20 CAR-Vδ1 γδ T cells exhibited innate and adaptive antitumor activities without the adverse event of xenogeneic graft-versus-host disease [236] occurring. Furthermore, both CD5- and CD19-NSCAR (non-signaling CARs) modified γδ T cells demonstrated a significant increase in killing T-ALL and B-ALL cell lines, respectively [237]. Although reports comparing the anti-tumor efficacy between CAR-γδ T cells and CAR-NK cells are not presently available, their distinct characteristics suggest differences in their applications and potential advantages in cancer treatment. These variances encompass cell type, recognition mechanisms, functions, origin, persistence, and potential side effects among γδ T cells, NK cells and their engineered counterparts.

## Strategies to overcome the limitations of CAR-NK cell therapy

Efforts are underway to pursue strategies aimed at overcoming the limitations of CAR-NK cell therapy, with the overarching objective of achieving potent efficacy and minimizing adverse effects. These strategies encompass the entire process, starting from CAR structure design to CAR-NK delivery and extending to the monitoring of efficacy and adverse effects. Three prominent topics in this endeavor are outlined below.

### Combinatorial antigen recognition to enhance target specificity and reduce off-target

Promising strategies are emerging to overcome these. Before discovering unique tumor-specific neoantigens, researchers explored logic gate inputs to enhance CAR-NK cell therapy specificity. In silico screening of 2.5 million dual antigens and 60 million triple antigens across 33 tumor types and 34 normal tissues, using Boolean logic gates like AND and NOT, revealed the potential of 2- to 3-antigen gates for improving CAR-T cell therapy specificity. Dual antigens notably outperformed single clinically investigated CAR targets, emphasizing their therapeutic promise [238]. CD147-synNotch-inducible GPC3-CAR-NK cells employ synNotch receptor to inducible the CAR expression to target a second tumor-related antigen GPC3 for highlighted specificity [226].

### Combination therapies to enhance antitumor efficacy and reprogram TME

Combination therapies have the potential to address the distinct challenges of engineered cellular therapies. Integrating cellular therapy with immune checkpoint blockade, bispecific antibodies, oncolytic virotherapy or small molecules have demonstrated enhanced antitumor activity [20]. Oncolytic adenoviruses (OAds) expressing TNF-α and IL-2 was able to induce CAR-dependent and CAR-independent host immunity and alter the immunosuppressive TME in pancreatic cancer [239]. CD19 CAR CB-NK cells with engineered IL-15 secretion were further enhanced through CISH knockout (KO) using CRISPR-Cas9 gene editing. This modification amplifies IL-15 signaling by overcoming inhibitory cytokine-related immune checkpoints. By targeting a cytokine checkpoint, this strategy reinforces the antitumor activity of IL-15-armored CAR-CAR-NK cells [240]. Soluble IL-15 and mIL-15 have been engineered into NK cells to enhance their survival, persistence, and activation in vivo. IL-15/IL-15Rα fusion protein (IL-15 superagonist) can bind to the intermediate-affinity receptor complex of IL-2R/IL-15R β- and γ-chains in the absence of cross-presentation by IL-15Rα on neighboring cells and exhibit its increased stability, improving the in vivo anti-tumor efficacy of CAR-NK cell therapy [241, 242].

It is recognized that immune checkpoints play pivotal roles in the immunosuppressive TME, contributing to NK cell exhaustion and facilitating tumor immune escape. These checkpoints may also induce exhaustion in CAR-NK cells, leading to resistance against tumors [243]. NK cells express checkpoint receptors such as PD-1, CTLA-4, LAG-3, TIM-3, TIGIT, NKG2A, and the Siglec family receptors (Siglec-7 and Siglec-9, CD200, and CD47). NKG2A is one of the most prominent inhibitory NK cell receptors. The combination of PD-1/PD-L1 blockers with CAR-T cell therapy has been shown to enhance antitumor activity and prevent T cell exhaustion in various studies [243]. This rationale may provide a basis for exploring combinational therapeutics reinvigorating CAR-NK cells [244].

Apart from gene engineering or genome editing of gene structures derived directly from NK cells, incorporating other genetic therapeutic elements or utilizing additional genetic therapeutic approaches should be considered in the context of CAR-NK cell therapy. CAR-NK cells undergo genetic manipulation to express specific targeting moieties, such as nanobodies or cell-penetrating peptides [245]. These targeting moieties are designed to selectively impact pathogenic cells or their microenvironments. The anti-ASC (apoptosis-associated speck-like protein containing a CARD) nanobody has demonstrated efficacy in disrupting pre-formed ASC oligomers, showing promise with treating inflammatory diseases in animal models [246]. The secretion of anti-ASC by CAR-NK cells has the potential to further enhance their anti-inflammatory efficacy, particularly in the context of inflammasome pathologies like Alzheimer’s disease.

### Optimization of CAR-NK manufacturing procedure

CAR-NK cells are obtained from various NK cell sources, necessitating tailored manufacturing optimization to produce “off-the-shelf” CAR-NK cell products with enhanced integrity, viability, functionality, and potency. This optimization spans NK cell isolation, culture and expansion, genetic manipulations and gene delivery, memory and persistence/longevity considerations, as well as preservation, post-thawing recovery, and administration protocols [31]. NK cell memory can be induced through the action of cytokines, exemplified by the generation of cytokine-inducible memory-like NK cells (MLNKs) and CAR-MLNKs with long persistence in vivo [19, 31, 176–179]. The ex vivo expansion of CAR-NK cells follows similar protocols to the ex vivo expansion of primary NK cells, requiring priming with cytokines to alleviate the potential for NK cell exhaustion and senescence [247, 248]. Maximal NK proliferation necessitates contact with NK-activated antigen-presenting cells (aAPC), activation of CD137 (4-1BB), and signaling through cytokines such as IL-2, IL-15, and IL-21, upon removal from maximally activating conditions, cytokines alone demonstrated the capability to sustain cytotoxic function against target cells [249]. Abnormality of the three-signal sequence in TME may cause impairment of CAR-NK cell persistence and functions. Evidence also indicates the detrimental effects of prolonged cytokine exposure in NK exhaustion-related dysfunction [244]. Current protocols for clinical-scale genome editing and CAR engineering of primary NK cells rely on feeder cell stimulation [31, 250]. Novel strategies for optimal feeder-free expansion should be adopted to streamline mass production of CAR-NK cells and ensure enhances in scalability and consistency in the manufacturing process [31]. Autologous CAR-NK cell therapy uses the patient’s own cells, reducing the risk of rejection and graft-versus-host-disease (GvHD). However, the process of harvesting, modifying, and expanding the patient’s own cells is both time-consuming and expensive. Allogeneic CAR-NK cell therapy offers an off-the-shelf solution, making it more accessible and scalable, but comes with some potential risks related to immune rejection and GvHD [251]. Advanced strategies under development include the exploration of in-vivo or in-situ CAR engineering and expansion, paving the way for the creation of living drugs with applications across a variety of diseases [252]. Given the development of a successfully streamlined manufacturing protocol with high yield and purity, along with similar characteristics to autologous CAR-NK cells, allogeneic CAR-NKT cells were demonstrated to have antitumor efficacy, expansion and persistence across various preclinical cancer models [253]. The initial clinical evaluation of allogeneic CAR-NKTs indicated objective response in the relapsed or refractory non-Hodgkin lymphoma (NHL) and acute lymphoblastic leukemia (ALL) patients with the well-tolerance. Allogeneic CAR-NKT may offer another promising candidates for ““off-the-shelf” cancer immunotherapy [254].

## Prospective future

NK cells play a unique role in antitumor responses through MHC-independent natural cytotoxicity, unique cytokine production, and immune memory. The emerging CAR-NK cell therapy shows promise in clinical research, displaying safety and preliminary efficacy in certain cancers. Despite distinct advantages over CAR-T cells, CAR-NK cells face challenges that necessitate careful consideration in their application and ongoing development. Efforts to enhance cell proliferation, improve cytotoxic activation, and optimize NK cell reconstitution with CAR structure and genomic engineering are paramount. Consequently, there is a pressing need for advancements in large-scale preparation methods, cryopreservation techniques, and overall efficacy. Addressing the challenge of the relatively short in vivo persistence and the potential for exhaustion represents an unresolved frontier in the field. CAR-NK cell therapy is positioned to be a versatile and advantageous treatment option, showing promise in various applications beyond cancer. The strong antitumor capabilities of NK cells form a solid foundation, and overcoming current challenges could lead to groundbreaking advancements in tumor treatment. The rapid evolution of NK cell-based immunotherapy, reflected in expanding cancer cell therapy pipelines, suggests that CAR-NK modifications will contribute to significant breakthroughs. In summary, the maturation of CAR-NK cell therapy technology in the future holds encouraging prospects for a broader range of cancer patients or other conditions, bringing us closer to addressing challenges in the treatment of refractory and recurrent cancer and other immune-mediated disorders.

## Data Availability

No datasets were generated or analysed during the current study.

## Abbreviations

CAR: Chimeric antigen receptor
2B4: CD244
4-1BB: Tumor necrosis factor ligand superfamily member 9
ACT: Adoptive cell transfer
ADCC: Antibody-dependent cell cytotoxicity
AICD: Activation-induced cell death
ALL: Acute lymphoblastic leukemia
AML: Acute myeloid leukemia
APC: Antigen-presenting cells
ASC: Apoptosis-associated speck-like protein containing a CARD
ASCT-1: Alanine/Serine/Cysteine/Threonine Transporter 1
ASCT-2: Alanine/Serine/Cysteine/Threonine Transporter 2
BaEV-LVs: Baboon envelope pseudotyped lentiviral vectors
BCMA: B cell maturation antigen
BPCDN: Blastic Plasmacytoid Dendritic Cell Neoplasm
c-Met: The hepatocyte growth factor receptor (HGFR)
CAR-NK cell: Chimeric antigen receptor-engineered NK cell
CAR-T cell: Chimeric antigen receptor-engineered T cell
Cas9: CRISPR associated protein 9
CB: Cord blood
CCL2: C–C motif chemokine ligand 2
CCR: Chimeric costimulatory receptors
CCR1: C–C Motif Chemokine Receptor 1
CCR2: C–C motif chemokine receptor 2
CCR5: C–C motif chemokine receptor 5
CCR7: C–C Motif Chemokine Receptor 7
CCRL2: C–C motif chemokine receptor like 2
CD3ζ: CD3 Zeta chain
CISH: Cytokine inducible SH2 containing protein
CLDX: Cell line derived xenografts
CLL: Chronic lymphocytic leukemia
CLTX: Chlorotoxin
CNS: The central nervous system
CR: Complete remission
CRISPR: Clustered regularly interspaced short palindromic repeats
CRS: Cytokine release syndrome
CS1: SLAM Family Member 7
CTLA-4: Cytotoxic T-lymphocyte-associated protein 4
CXC3CR1: C-X3-C motif chemokine receptor 1
CXC6: C-X-C motif chemokine receptor 6
CXCL1: The chemokine (C-X-C motif) ligand 1
CXCL10: The chemokine (C-X-C motif) ligand 10
CXCR3: C-X-C motif chemokine receptor 3
CXCR4: C-X-C motif chemokine receptor 4
CXCR6: C-X-C Motif Chemokine Receptor 6
DAP12: DNAX-activation protein 12
DC: Dendritic cells
DLBCL: Diffuse large B-cell lymphoma
DLL3: Delta Like Canonical Notch Ligand 3
DNAMI-1: DNAX accessory molecule-1
EBNA3C: Epstein-Barr virus nuclear antigen 3C
EF1α: Elongation factor 1-alpha promoter
EGFR: Epidermal growth factor receptor
EGFRvIII: The epidermal growth factor receptor variant III
EOMES: Eomesodermin
EpCAM: Epithelial cell adhesion molecule
ErbB2: Erb-B2 Receptor Tyrosine Kinase 2
ETS1: ETS proto-oncogene 1
FAP: Fibroblast activation protein
FcγR: Fc-gamma receptor
FDA: US food and drug administration
FLT3L: FMS-like tyrosine kinase 3 ligand
FOXO3: Forkhead Box O3
GFP: Green fluorescent protein
GM-CSFRα: Granulocyte–macrophage colony-stimulating factor receptor α chain
GMP: Good manufacturing practice
GPC3: Glypican-3
GvHD: Graft-versus-host disease
HAVCR2: Hepatitis A virus cellular receptor
HCC: Hepatocellular Carcinoma
HD: Hinge domain
HER2: Human epidermal growth factor receptor 2
HLA: Human leukocyte antigen
HLH: Hemophagocytic lymphohistiocytosis
hnCD16: High-affinity, non-cleavable CD16
HNSCC: Head and neck squamous cell carcinoma
hPGK: Human phosphoglycerate kinase promoter
iCAR: Inhibitory CAR
ICD: Intracellular domain
ICIs: Immune checkpoint inhibitors
ICOS: Inducible T Cell Costimulator
IDO: Indoleamine 2,3-dioxygenase
IFNAR: IFN-Alpha/Beta Receptor 1
IFNβ: Interferon beta
IL-12: Interleukin 12
IL-12R: Interleukin-12 receptor
IL-13Rα2: Interleukin 13 receptor alpha 2
IL-15: Interleukin 15
IL-15R: Interleukin-15 receptor
IL-18: Interleukin 18
IL-18R: Interleukin-18 receptor
IL-1R: Interleukin-1 receptor
IL-2: Interleukin 2
IL-21R: Interleukin-21 receptor
IL-2R: Interleukin-2 receptor
IL-4R: Interleukin-4 receptor
iPSC: Induced pluripotent stem cell
IRF7: Interferon Regulatory Factor 7
ITAM: Immunoreceptor tyrosine-based activation motifs
KDR: Kinase insert domain receptor
KIR: Nature killer Ig-like receptor
KIR2DS2: Killer-Cell Immunoglobulin-Like Receptor Two Domains Short Tail 2 Protein
KO: Knockout
LAG-3: Lymphocyte-Activation Gene 3
LFA1: Lymphocyte Function-Associated Antigen 1
LUAD: Lung adenocarcinoma
LV: Lentivirus
MAS: Macrophage activation syndrome
mb: Membrane-bound
mbIL-15: Membrane-bound interluekin-15
mbIL-21: Membrane-bound interluekin-21
MDS: Myelodysplastic Syndromes
MDSC: Myeloid-derived suppressor cells
MHC: Major histocompatibility complex
MICA: MHC class I polypeptide–related sequence A
MICB: MHC class I polypeptide–related sequence B
ML: Memory-like
MLNKs: Memory-like NK cells
MM: Multiple myeloma
mPGK: Mouse phosphoglycerate kinase promoter
Muc1: Mucin 1
NCRs: Nature cytotoxic receptors
NF-κB: Nuclear Factor Kappa B Subunit
NHL: Non-Hodgkin lymphoma
NK cell: Natural killer cell
NKG2A: NK cell receptor A
NKG2C: NKG2-C-Activating NK Receptor
NKG2D: NK Cell Receptor D
NKG2E: NKG2-E-Activating NK Receptor
NKp44: Natural Killer Cell P44-Related Protein
NKp46: Natural killer cell P46-related protein
NKp80: Natural Killer Cell P80-Related Protein
NKPp30: Natural Killer Cell P30-Related Protein
NSCLC: Non-small cell lung cancer
OX40: TNF Receptor Superfamily Member 4
PB: Peripheral blood
PCL: Plasma cell leukemia
PD-1: Programmed cell death protein 1
PD-L1: Programmed death-ligand 1
PDX: Patient-derived xenografts
PFS: Progression free survival
PGE2: Prostaglandin E2
PSCA: Prostate stem cell antigen
R/R: Relapsed or refractory
RD114: RD114 envelope protein
ROBO1: Roundabout Guidance Receptor 1
RRMM: Relapsed/refractory multiple myeloma
S1P5: Sphingosine-1 phosphate receptor 5
scFv: Single-chain variable fragment
SCLC: Small Cell Lung Cancer
SFFV: The spleen focus-forming virus
Siglec-7: Sialic acid binding Ig like lectin 7
Siglec-9: Sialic acid binding Ig like lectin 9
SLAMF7: SLAM Family Member 7
SLE: Systemic lupus erythematosus
SP: Signal peptide
T-BET: T-box transcription factor
TAM: Tumor-associated macrophages
TCR: T cell receptor
TGF-β: Transforming growth factor beta
TIGIT: T cell immunoreceptor with Ig and ITIM domains
TIM-3: T-cell immunoglobulin mucin family member 3
TLR2: Toll-Like Receptor 2
TMD: Transmembrane domain
TME: Tumor microenvironment
TNBC: Triple-negative breast cancer
TNF: Tumor necrosis factor
TNFRSF: The tumor necrosis factor receptor superfamily
TRE: Target recognition element
Treg: Regulatory T cells
TROP2: Trophoblast cell surface antigen 2
TSA: Tumor-specific antigen
VH: Variable region of heavy chain
VL: Variable region of light chain
VSVG: Vesicular stomatitis virus G protein
ZAP70: Zeta Chain Of T Cell Receptor Associated Protein Kinase 70
αFR: Folate receptor alpha
γδ T cell: Gamma delta T cell
ΔIL2RB: Truncated IL-2 receptor beta
